# Elephant Endotheliotropic Herpesvirus Impact in the European Asian Elephant (*Elephas maximus*) Population: Are Hereditability and Zoo-Associated Factors Linked with Mortality?

**DOI:** 10.3390/ani11102816

**Published:** 2021-09-27

**Authors:** Sónia A. Jesus, Marcus G. Doherr, Thomas B. Hildebrandt

**Affiliations:** 1 Department of Reproduction Management, Leibniz Institute for Zoo and Wildlife Research, Alfred-Kowalke-Straße 17, 10315 Berlin, Germany; hildebrand@izw-berlin.de; 2 Institute for Veterinary Epidemiology and Biostatistics, Freie Universität, Königsweg 67, 14163 Berlin, Germany; marcus.doherr@fu-berlin.de; 3 Faculty of Veterinary Medicine, Freie Universität, Oertzenweg 19 b, 14163 Berlin, Germany

**Keywords:** EEHV, *Elephas maximus*, epidemiology, hemorrhagic disease, hereditary, proboscivirus, zoological institution

## Abstract

**Simple Summary:**

Elephant Endotheliotropic Herpesvirus hemorrhagic disease (EEHV-HD) is considered the primary cause of calf mortality in the global captive Asian elephant population. Once thought to be exclusively a zoo problem, EEHV-HD is currently acknowledged as a disease also present in wild populations, although the extension of this threat in some free-range countries is still poorly understood. The disease is characterized by an acute hemorrhagic syndrome due to vast endothelial destruction combined with disseminated intravascular coagulation, leading to the sudden death of mainly young elephants. In this study, we aimed at understanding the impact of EEHV-HD in the European captive Asian elephant population and acquiring a better understanding if hereditary or environmental factors could be linked to the manifestation of this disease. The findings of this investigation suggest the involvement of zoo-associated factors with possible sire or dam (or a combination of both) influence on the onset of the disease. This knowledge points us to the importance of continuous retrospective epidemiological studies and stresses the great importance of finding further underlying factors for the development of this disease if we wish to halt the high number of deaths caused by this hemorrhagic disease.

**Abstract:**

EEHV is a ubiquitous virus, which most likely has co-evolved with elephants and is shed by healthy individuals and maintained in the herds. Yet, the factors determining calf susceptibility to the virus remain unknown. Here, we explored the impact of EEHV-HD in the European captive Asian elephant population in a retrospective statistical study spanning the last 35 years. We show that EEHV-HD was implicated in more than half of all deaths recorded in calves older than one months old. Moreover, the median age across EEHV-HD fatalities was significantly lower compared to other death causes. Finally, we investigated if heredity and zoo-associated factors could be linked to a higher susceptibility of calves to this disease. We used a univariable logistic regression model to evaluate if either fathers, mothers, or zoos could, separately, be considered as risk factors to the development of the disease. Afterwards, we used a two multivariable model, combining: (1) fathers and zoos, and (2) mothers and zoos. Overall, we found that two fathers, one mother, and four zoos had three or more times higher risk of their calves becoming sick when compared to all others, pointing us to the presence of a management or environmental element, which can have paternal and maternal influence and leads to calf susceptibility or resistance to EEHV-HD.

## 1. Introduction

Elephant Endotheliotropic Herpesvirus (EEHV) was initially reported in the captive Asian elephant population in 1990 after a three-year-old elephant calf died from an acute hemorrhagic disease (HD) [1]. At necropsy, a severe generalized hemorrhagic condition due to vascular endothelial lesions was observed [1]. Diseased elephants experience a rapid and systemic spread of the virus, followed by vascular endothelial cell damage associated with an uncontrolled virus replication [2,3]. This fulminant disease affects mainly very young calves, often leaving little or no time to provide adequate veterinary treatment [4,5]. Multiple EEHV genotypes and strains have been reported, with EEHV 1 being the most impactful [6,7,8,9]. In the European population, 80% of the calves’ EEHV-related deaths were reportedly caused by subtype EEHV1a [10].

EEVH-HD is considered to be an ancient infection among Asian elephants {Formatting Citation} and is not a disease exclusive of this species as it may also affect African elephants. However, the recorded mortality rate in African elephants is lower, and the animals seem to present symptoms at an older age [11,12,13]. Currently, the most used antiviral treatment is a human anti-herpetic drug, despite its high costs and reported as presenting unproven efficacy, so far [4,14,15].

Once thought to be an exclusive zoo disease, fatal cases due to EEHV-HD have been reported in several range countries, such as India [9,16], Thailand [2,6,17], Cambodia [18], Laos [19], Myanmar [8], Nepal, and Sumatra [7]. The prevalence of EEHV-HD in wild populations is expected to be high, since the medical veterinary teams working in close association with these populations have found evidence of this disease, during necropsies. However, due to a lack of logistic capacities, further investigations have been hampered [20]. In North American zoos, reports show that 53% of deaths since 1980 in their Asian elephant population were caused by EEHV-HD, while in Europe this accounts for 60% of the total deaths since 1995 [20]. Additionally, North American institutions reported that the virus presents a mortality rate of 68% [20]. In 2016, 40% of elephants’ deaths in the UK and Ireland were caused by EEHV-HD with an overall population mortality of 21.6% [4], making this the major mortality cause in both continents [20]. In range countries, such as India, a prevalence study showed that at least one of the EEHV variants is present in 35% of their captive Asian elephants [21]. Moreover, in Thailand, a seroprevalence of 42% was found (in private, touristic, and logging elephant camps [22], showing that EEHV is also maintained within the captive population. Most infectious diseases run a subclinical course and only part of the population will present clinical disease, where the mutual interactions between environment, host, and pathogen genetic factors, influence this ratio [23]. To similarity, EEHV-HD must also be influenced by the elephant host genetics and environmental pressures, being the presence and pathogeny of the virus alone, not the only determinant factor.

Even though this disease has been under study for the past three decades, and a significant number of discoveries were recently made on its pathophysiology [2,24], the adequate treatment, and the epidemiological impact of it in the overall world elephant population is still not fully understood. Therefore, having a deeper understanding of the virus’ mechanism of action is yet of the highest priority. Moreover, there is an urgent need to identify what risk factors are involved in the onset of the disease, in order to establish proper actions to protect the calves.

This study aims to assess the impact of EEHV-HD in the European captive Asian elephant population and to explore risk factors linked to a higher prevalence of the disease, such as gender, age, genetic lineage, and location. To address these, we used historical and current data from all captive calves born in Europe from January 1985 to June 2020, conducting the longest, retrospective, and longitudinal observational study so far. The disease seems to affect calves from different genetic backgrounds and breeding facilities at a different rate: while some are profoundly impacted by this hemorrhagic disease, others are minimally or not affected. Therefore, we hypothesize that hereditary (host genetics) and different zoo-associated factors (e.g., management protocols and growing environment) may protect calves against the potentially fatal outcome of the disease.

## 2. Materials and Methods

### 2.1. Data Collection

To be able to identify the impact of EEHV-HD, regardless of the virus genotype, in the captive-born Asian elephant population in Europe and investigate the risk factors associated with high mortality, we compiled a dataset of all animals kept in captivity at European zoos, spanning the last 35 years, from January 1985 to June 2020 (*n* = 330, supplementary materials, Table S1—Study population database). This dataset comprises exact birth and death dates, maternal and paternal information, location, and the present status of the elephants (alive, dead by other causes, or dead by EEHV-HD), as well as EEHV infection reports. We collected information from the Asian elephant European Association of Zoo and Aquaria ex situ Programme (EEP, formerly European Endangered Species Programme) Studbook yearly reports, from Zoological Information Management system (ZIMS), from personal contacts with the zoological institutions, from up-to-date registers documented on zoo websites, and from information compiled at elephant large online databases.

### 2.2. Data Cleaning, Selection and Analysis

The starting year of the analysis (1985) was chosen to match the year when the first reported EEHV-HD fatal case was born—Lohimi, a female calf born in a circus, that presented a hemorrhagic syndrome in 1988, which led to her death, at the age of three years [1]. Since the population in the study were captive European Asian elephants, only calves born in captivity were kept in the data set, and all wild-born animals were removed from the study. Thus, non-European captive calves that were translocated to Europe afterwards were also not considered for analysis.

Neonatal mortalities and early life deaths accounted for 24.8% of the total deaths due to several causes (e.g., miscarriages, abortions of twinning, stillbirths, surgically removed fetuses, infanticide, rejected by the mother). On this account, a subset of our initial population was created, including only records of successful births and minimal management to ensure a correct adaptation to the first months of life (e.g., proper feeding and non-life-threatening congenital defects). Animals that did not survive to reach two months of age (*n* = 83; *n* = 77 under one week and *n* = 6 dying in their first month of life) were excluded from this dataset. Under this threshold, three animals were mentioned as possible EEHV-HD deaths, presenting low titters of the virus, being stillborn, or having succumbed under 24 h after parturition. These deaths could not be clearly attributed to EEHV-HD and were removed.

The frequencies of births, deaths due to EEHV-HD, and deaths due to other causes per year of study are shown and their distributions were evaluated. We investigated the trends of distribution according to age for each status (status 0 = alive, status 1 = death by EEHV-HD, and status 2 = death by other causes) for all captive-born elephants. Standardized residuals were visually assessed and were not fully normally distributed, therefore, a non-parametric Kruskal–Wallis test was used to compare median ages between groups.

The association of gender with the overall survival time for the entire population in the study and within the EEHV-HD reported cases was investigated using the log-rank (Mantel–Cox) test. Afterwards, a survival analysis (Kaplan–Meier curve) was performed to compare the survival time between the animals that presented EEHV-HD disease (that survived or died) and all others that never presented symptoms.

Finally, to test if hereditary lineage and/or the environment could be potential risk factors to the survival of the elephants in captive populations, we categorized all calves by fathers, mothers, and location during calfhood. The identities of the bulls, dams, and zoos will remain anonymous in our study.

An explorative univariable logistic regression model was performed to separately assess the odds ratio (OR) of EEHV symptomatic calves for individual (i) fathers, (ii) mothers, and (iii) zoos. Parents and zoological institutions included in the analysis were grouped according to the number of calves produced. Bulls that sired more than ten calves were kept individually while all bulls that sired fewer calves were collapsed into a single group (bulls that sired less than ten offspring). For dams and zoos, the cut-off for keeping them individually was five calves. Fathers, mothers, and zoos with a lower number of calves were considered the baseline for comparison. Afterwards, two multivariable models estimated simultaneously the OR of (i) fathers and zoos as well as (ii) mothers and zoos combination. Results were screened for OR greater than 6.0 which indicates a sixfold higher chance of presenting EEVH sick calves when compared to the baseline category.

All statistical analyses were performed considering an alpha level for significance and tendency of 0.05 and 0.10, respectively. Analyses were conducted using IBM SPSS (IBM SPSS Statistics for Windows, version 24.0, Armonk, New York, NY, USA) predictive analytics software and graphs were produced using GraphPad Prism (version 9, GraphPad Software, San Diego, CA, USA).

## 3. Results

### 3.1. Descriptive Analysis

A total of 247 captive-born Asian elephants (females = 116, males = 131) were born between January 1985 and June 2020 and survived more than one month of life. These births occurred in a total of 48 European zoological institutions and animals are now distributed in 68 zoological locations, due to transfers between zoos. A total of 72.1% of the population monitored since 1985 never presented the disease and are still thriving at the moment of writing. We found that 15.8% (*n* = 39) of the calves were infected and symptomatic for EEHV-HD. Of this percentile, 13.4% were lost to the hemorrhagic disease, and therefore, so far, only 2.4% (*n* = 6) of the affected calves managed to resist and survive this disease.

A total of 25.5% (*n* = 63) of the population died within the study period due to several different causes (e.g., foot disease, infectious diseases—including EEHV-HD, tumors, etc.). Accordingly, EEHV-HD is the primary cause of death above one month of age in the European Asian elephant population, producing 52.5% of all reported deaths (*n* = 33).

**Figure 1:**
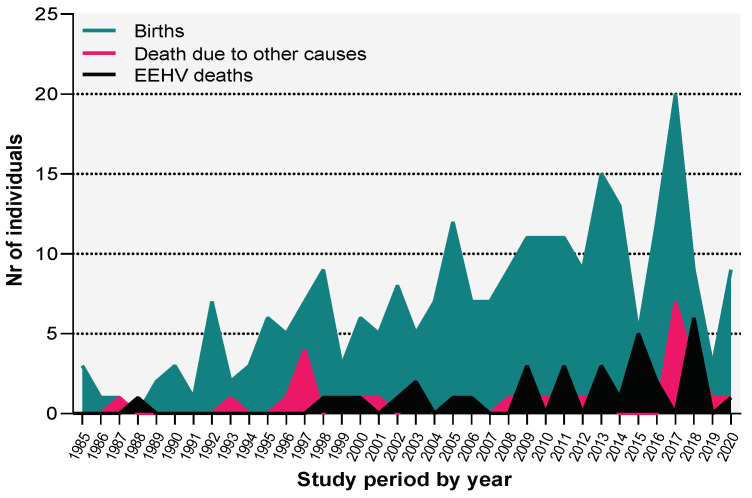
Distributions of the births (green), deaths unrelated (red) to, and related to EEHV-HD (black) of the captive-born Asian elephant calves above one month of age, in the European population from 1985 to 2020.

We found that only in 1988 no births were registered, and one death was reported, presenting, therefore, a negative balance for that specific year. Moreover, except for 1987, 2015, and 2018 where the number of births and deaths was the same, the number of offspring per year exceeds the number of deceased animals (Figure 1).

### 3.2. Survival Age and Gender Relation

There was no impact of gender in the survival time of Asian elephants born after 1985 in Europe (*p* = 0.813) and EEHV-HD fatalities were also not gender related, with an almost 1:1 relationship (females *n* = 17, males *n* = 16). Moreover, males (*n* = 131) were found to be younger than females (*n* = 116); the overall male median age was around 24 years of age while the female average rounded 30 years.

**Figure 2:**
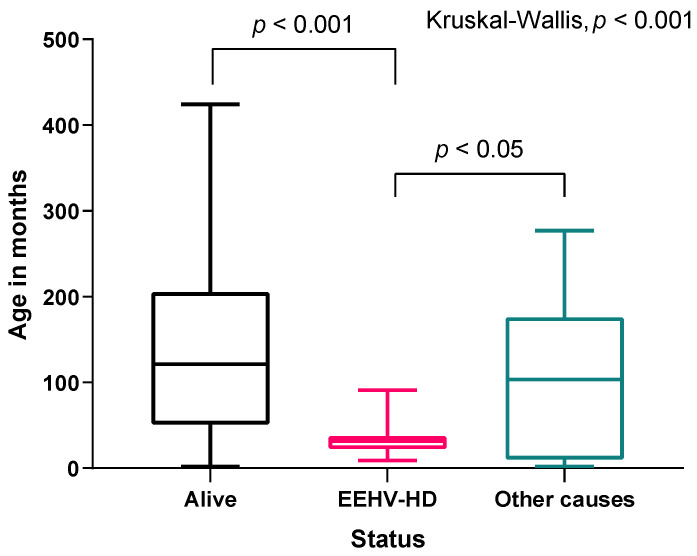
Boxplot representation of the overall survival time distribution of the calves in months, for the living animals, deaths caused by EEHV-HD, and deaths due to other causes. The box represents the 25th to 75th percentile values of the distribution (interquartile Range), the line within the box the median (50th percentile), and the whiskers approximate the 2.5th and 97.5th percentile values.

The animals which died from various non-EEHV-HD-related causes (*n* = 30), lived between two months and 23 years (median = 8.6 years). For the EEHV-HD fatal cases (*n* = 33), the earliest related death occurred at 9 months old, and the oldest animal died at 7.6 years of age resulting in a very narrow age range. Additionally, deaths due to this virus occurred at a significantly lower median age (2.7 years old) when compared to the median age of elephants that died due to other causes (8.6 years old) (Figure 2).

Pairwise comparisons between EEHV fatal cases and animals that are alive revealed a significantly lower age of life for the diseased animals (*p* < 0.001). The same results were found for the comparison between animals dead due to other causes and those that succumb to EEHV (*p* = 0.007). The median age did not differ between the living animals and those that died due to other causes (*p* = 0.057).

**Figure 3:**
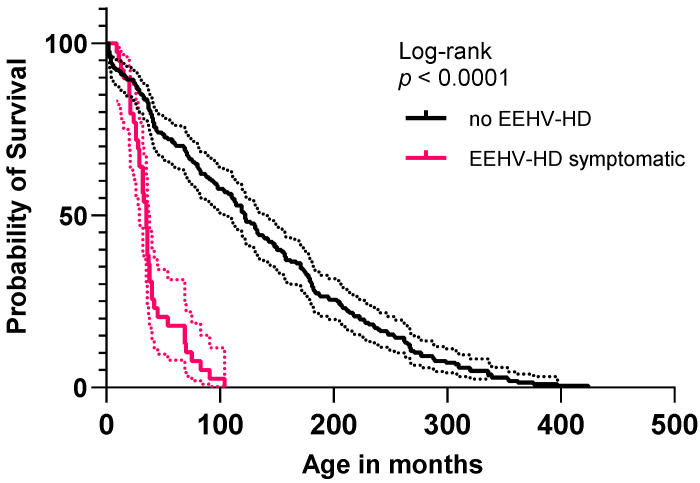
Kaplan–Meier survival curves, distributing the age of infected animals that presented the disease (median 35 months) and the age of the other population in the study (median 122 months). *p* values obtained using the log-rank test show *p* < 0.0001.

Kaplan–Meier analysis revealed that the survival curve of the animals that presented EEHV-HD and the survival curve of the other individuals that never presented symptoms are significantly different (*p* < 0.001, Figure 3). The median survival age of EEHV-HD symptomatic animals was 35 months, while animals with no reported EEHV-HD presented a median age of 122 months.

### 3.3. Father and Mother Distribution of EEHV-HD Fatal Cases

**Figure 4:**
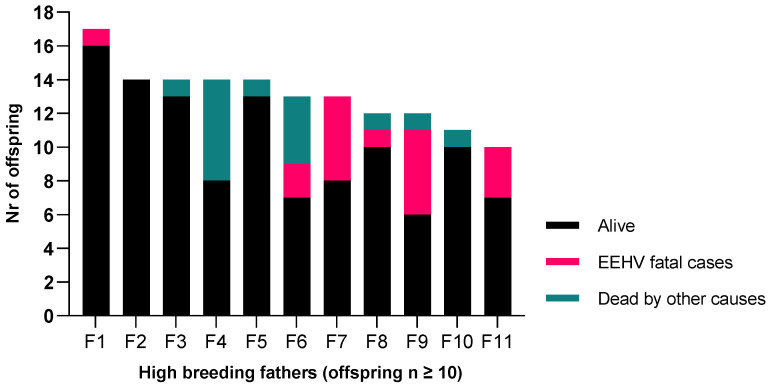
Distribution of the offspring which are still alive, have died due to EEHV-HD, or have died by other causes, per high breeding fathers (*n* = 11, each producing ten or more calves).

When investigating the distribution of the fatal EEHV cases per high breeders, we found that some fathers presented no loss of their offspring due to EEHV-HD (e.g., fathers F2, F3, F4; Figure 4) or minimal loss (e.g., father F8, Figure 4), while others, with nearly the same number of calves, have lost a high percentage of their calves (e.g., fathers F9 with 42% and F7 with 38% of calf loss due to EEHV-HD; Figure 4).

From all the fathers analyzed (*n* = 45), 11 bulls had ten or more calves each. These animals have produced nearly 60% (*n* = 144) of the entire population present in the study population and were the ones used for the subsequent analysis of parental risk. Calves born to two specific fathers with a high frequency of offspring presented a significant increase associated risk to present EEHV-HD (F7, OR = 3.8, *p* = 0.03; F9, OR 4.4, *p* = 0.02) when compared with other sires.

Maternal contribution (*n* = 97) to the overall deaths of the calves was also investigated; however, there is a very low frequency of births registered per dam when compared with the high offspring number presented by the fathers. One mother presented an increased tendency for her calves to have the disease when compared to all other mothers in the study (OR = 3.8, *p* < 0.1).

### 3.4. Zoo Distribution of EEHV-HD Fatal Cases

Our survival comparisons based on the living location (*n* = 68 zoos) of the calf showed that high breeding zoos (*n* = 18) that produced five or more calves conceived a total of 140 calves. The remaining 50 locations presented a lower breeding rate and produced 107 offspring, with the majority of the zoos having produced one or two calves.

**Figure 5:**
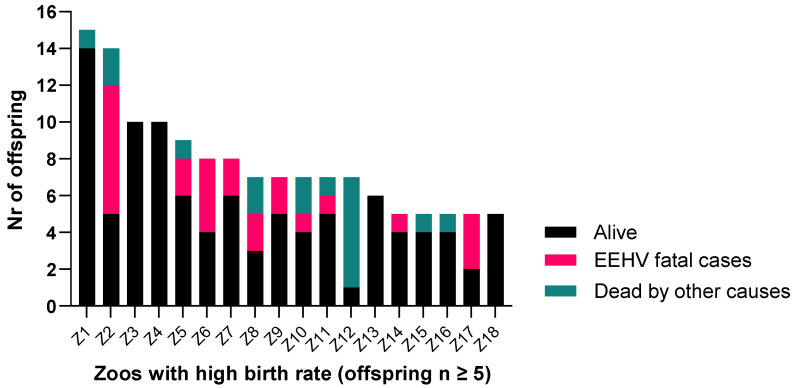
Distribution of offspring that are still alive or died due to EEHV-HD or other causes, by zoos (*n* = 18) that have produced five or more calves during the study period.

Similar to the distribution found for the fathers, we observed that some institutions have suffered high losses. When investigating only the zoos that bred five or more times, we found that some of these locations were not affected at all, while others present an overall offspring loss due to EEHV as high as 50% of the total offspring born at a particular zoo (e.g., zoos Z11 and Z6; Figure 5).

We found that three institutions presented a significantly increased odds ratio, between 8 to 12 times higher, for their calves to present EEHV-HD (Z17, OR 11.8, *p* = 0.01; Z2, OR 10.6, *p* < 0.001; Z6, OR 7.9, *p* = 0.007), than the other zoos in the study.

In the multivariable model with fathers and zoos, we found that F9 and Z17 presented a significant increased OR for presenting calves with the disease (OR 6.2, *p* = 0.04 and OR 19.1, *p* = 0.016, respectively) and Z9 had an OR > 6.0 (*p* = 0.086). When combining with mothers, we find that zoos Z2, Z6, and Z17 present a significantly higher probability of reporting calves with EEHV-HD (OR > 6.0, *p* = 0.012, *p* = 0.002, and *p* = 0.013, respectively).

On another analysis, a cross-tabulation of all fatal cases caused by EEHV-HD by the respective fathers (*n* = 18) and locations (*n* = 18) showed deaths attributed to different sires at the same zoo. Likewise, different calves fathered by the same sire but living in different institutions were also lost (Supplementary Materials, Table S2—Crosstabulation of the distribution of EEHV-HD fatal events per Father and Zoo, for the captive European Asian elephant). At the end of the study, there were 18 calves reaching, or near the age of 2.7 years, the statistical age risk to succumb to EEHV-HD.

## 4. Discussion

In the present study, we compiled all data available since the first detection of a captive Asian elephant with EEHV-HD was detected, making it the most extensive study on the impact of EEHV on the European Asian elephant population to date. Our data showed that EEHV-HD affected calves at around 2.7 years old, which is significantly lower than the median age for other causes of death (8.6 years). These results are in accordance with recent reports from Europe, Thailand, and North American risk ages [6,10,20]. The European Endangered Species Programme’s latest report states that birth rates will not replace the loss of the high number of aged females (35–55 years old), of which the majority is considered unable to further reproduce. This will possibly lead to a decrease in female captive elephants in the future. The report also suggested that female elephants should become pregnant for the first time at 8 years of age and that ideally, there is an interbirth interval of 7 years [25]. Since EEHV-HD deaths occur at a significantly lower and narrower age range than other causes, killing mainly youngsters before sexual maturity is reached will, therefore, reduce the possibility of these calves substituting the elder ones, as well as reducing the overall number of possible future breeders. Consequently, this affects the breeding efforts made by the zoos on keeping a reproductive group to maintain a healthy and sustainable captive population.

After removing all premature deaths, EEHV-HD alone was responsible for 52% of fatalities, nearly the same amount as reported for North American institutions (53% [20]). This mortality rate for EEHV deaths in Europe is slightly lower than the previous study published for the continent (57%—for calves surviving the first day of life, data until 2017; [10]) and is most likely a reflection on the increased number of survivors and the outstanding birth rate that year (20 new births in 2017). Despite the similarities between different countries, when we compare mortalities, we found that EEHV-HD presented a higher mortality rate in the European population (85%), compared to the one reported for North America (68%; [20]) or Thailand (nearly 69%; [6]). There are no indications that a more virulent serotype of the virus is present in Europe, therefore it is most likely that this mortality rate difference is related to the management of the disease. Due to their tradition of elephant training under the guidance of the mahouts, Thailand has facilitated veterinary access to these animals, to perform medical check-ups, and to treat very young calves. In the North American captive population, although in a protective contact system (where elephant keepers must not share the same unrestricted space with elephants), their management allows for direct training of young calves up to 24 months of age [26]. This allows for regular monitoring, as well as prompt and more effective prevention or veterinary treatment of calves once symptoms are present. In Europe, all EAZA members must also comply with the protected contact handling policy as it will become effective from 2030 on [27]. European zoos are also encouraged to start training their calves from the age of 4 months, and several behaviors facilitating medical support are expected to be achieved by the age of one year, for all breeding European institutions [14]. Hence, the lower mortality rates presented by Thailand and North America most probably reflect the substantial amount of survival cases due to effective treatment when compared to the population of this study. Nevertheless, the numbers of survivors in Europe have risen in the past few years, and it is expected to improve due to a higher awareness of the disease and the positive outcome that early monitoring and fast medical intervention can have.

In this study, Europe presented lower EEHV-HD morbidity (15.8%) than North America, where one in every four calves (25%) has presented the disease [20]. This finding suggests that the European captive-born calves, although also exposed to the virus, become ill with EEHV-HD less often. However, it is most likely that this is related to under-detected or subclinical cases during the past years in Europe.

Bennet [28] has performed a genogram on the Asian elephant captive populations living in Europe and North America to assess the possibility of a family link and found that EEHV-HD-related deaths appeared to be grouped into clusters. However, since the elephants in that study were originally located at the same institution, it remained unclear whether the clustering was due to genetic or environmental pressure [28]. Our study supports this indication of clustering of cases in certain zoos and accepts potential effect modification by either mothers or fathers. Combining a multivariable model, fathers and zoos revealed a higher risk for two specific zoos and one father to have their calves developing the disease. In the model using mothers and zoos, we also find a significantly higher risk for three specific zoos. Together, these findings indicate the possibility of a multifactorial disease, where a zoo-associated component must be assumed to be involved and a hereditary predisposition might be expressed under the influence of certain environmental pressures. This highlights the importance of collecting relevant risk factor information for all calves (retrospectively and prospectively) for more detailed analyses on risk factors. As an example, a hereditary coagulation disorder has been reported in an Asian elephant herd, where a breeding bull, although asymptomatic, presented a prolonged prothrombin time (one of the tests used to assess coagulation capability). This coagulopathy was caused by a specific mutation, leading to a lack of activity of one important clotting factor (coagulation factor VII) which led to an increase in bleeding time. Three of his five offspring were reported to be carriers of this mutation [29]. How the body of a calf, carrier of this hereditary coagulopathy, would react to the vascular endothelial damage caused by EEHV is unknown.

One can debate whether the initial year of the study (1985) may be considered premature since, in the ’80s, diagnostic techniques for EEHV were not sufficiently developed or accurate. Part of the diagnostic gaps in the early years of the study period have been addressed by performing retrospective analyses with qPCR in frozen samples. These samples were tested to detect and quantify the virus, giving us a better idea of possible past cases that might have been overlooked [9,18,30].

The narrow age range of EEHV-HD deaths found also implies that there might be an essential element that debilitates the Asian elephant calves at this specific age of their life. Therefore, a stressful element may play a part in triggering the virus, but also, there might be protective factors helping the calves that survived this risk age to overcome this period and thrive. Therefore, another worthwhile line of research would be to focus on finding what are the protective risk factors, especially at this sensitive young age.

EEHV should not stop breeding programs at zoological institutions due to several reasons, including the continuous decrease of the global population of Asian elephants and its endangered status to face extinction. Lethal cases of this disease are found worldwide, and reports show that EEHV is ubiquitous and that elephants are the natural host and co-evolved with EEHV [5,9]. It is essential to gain a better knowledge of the disease’s pathophysiology and risk factors, to support the development of vaccination, and to improve treatment. All these research efforts to deepen this virus’ investigations can only be undertaken at a global scale and they are of extreme importance to halt EEHV-HD.

At the end of this study, 80 Asian elephant calves were at the age of the previously reported fatal cases. Therefore, routine monitoring of these young calves and preparedness to tackle this disease is crucial to favor a positive outcome of the disease, while efforts to find more epidemiological risk elements of this hemorrhagic disease should be under investigation.

Finally, this is an observational study, and therefore it is not possible to prove causality. Nevertheless, it guides us on the importance of follow-up studies to assess management conditions and to find the factors that protect or place the calf at a higher mortality risk. It is important to mention that the most meaningful and novel findings of this statistical study come from the updating and continuous analysis of a long-life living being, with a very long gestation time and inter-generational gap, enlightening the importance of longitudinal studies in elephants. Therefore, we suspect that more fathers, mothers, and institutions will be considered as related risk factors in the future and suggest that the starting period of this study should be used as a “milestone” for further studies.

## 5. Conclusions

This longitudinal epidemiological study investigates the elephant endotheliotropic herpesvirus impact in European zoological institutions, using the largest up-to-date dataset on captive Asian elephants.

Our findings support previous studies, showing that EEHV is the primary cause of death among Asian elephants, besides neonatal mortality. Calves with EEHV-HD died at a very young age, around 2.7 years old (median age), which is a significantly younger age at death than that for other causes. Nevertheless, it is important to keep monitoring for EEHV until a later age of at least 8 years old (the oldest animal died with EEHV at 7.6 years of age).

The results of this study suggest the involvement of zoo-associated factors, which might in part be related to management, and which can be influenced by either father or mother (or a combination of both), on the onset of EEHV-HD. Indeed, in total, two fathers, one mother, and four zoos presented a higher risk for their calves to develop the disease, when compared to all others in the study, hinting at the involvement of one or more environmental and triggering elements, with possible genetic associations.

More focus needs to be placed on the underlying factors of this disease, in particular, the study of management differences between zoos with a higher risk of fatal outcomes due to EEHV-HD and low-risk zoos could inform Studbook breeding decisions.

## Data Availability

Not applicable.

## Supplementary Materials

The following are available online at https://www.mdpi.com/article/10.3390/ani11102816/s1, Table S1: Study population database, from January 1985 until June 2020, Table S2: Crosstabulation of the distribution of EEHV-HD fatal events per Father and Zoo, for the captive European Asian elephant.
